# Natural killer cells are recruited during pulmonary tuberculosis and their *ex vivo* responses to mycobacteria vary between healthy human donors in association with KIR haplotype

**DOI:** 10.1111/j.1462-5822.2012.01834.x

**Published:** 2012-07-30

**Authors:** Damien Portevin, Laura E Via, Seokyong Eum, Douglas Young

**Affiliations:** 1 Division of Mycobacterial Research MRC National Institute for Medical ResearchThe Ridgeway Mill Hill London NW7 1AA UK; 2 Tuberculosis Research Section LCIDNIAID, NIH 33 North Drive Bethesda MD 20892 USA; 3 International Tuberculosis Research CenterGapo-dong Masan Changwon Korea

## Abstract

Humans vary widely in their susceptibility to tuberculosis. While only a minority will progress to disease, the majority of healthy individuals exposed to *Mycobacterium tuberculosis* mount an immune response that can clear or contain the infection in a quiescent form. Using immunofluorescence on human clinical samples, we identified natural killer (NK) cells infiltrating granulomatous pulmonary lesions during active disease. In order to compare the NK cell ability to react to free mycobacteria in the context of tuberculosis infection and *Mycobacterium bovis* BCG vaccination, NK cells were isolated from the peripheral blood of anonymous healthy human donors, and stimulated with *M. tuberculosis* H37Rv or *M. bovis* BCG. Extracellular *M. tuberculosis* and *M. bovis* BCG could equally trigger the release of IFNγ and TNFα from NK cells in the presence of IL-2. However, we found that this response varied 1000-fold between individuals (*n* = 52), with differences in KIR haplotype providing a significant criterion to distinguish between low and high responders. Our findings suggest that variations at the KIR locus and therefore of the NK cell repertoire may affect cytokine production in response to mycobacteria and we propose that this innate variability couldsustain different levels of susceptibility to *M. tuberculosis* infection.

## Introduction

Aerosol transmission of *Mycobacterium tuberculosis* during active pulmonary disease results in exposure of a substantial proportion of the global population, although only a fraction of individuals develop clinical tuberculosis (de Jong *et al*., 2008). Based on priming of an antigen-specific immune response, it is estimated that two billion people have been infected by *M. tuberculosis*, of whom 5–10% will go on to develop disease (WHO report, updated annually). Epidemiology studies suggest that around 20% of individuals intensively exposed to *M. tuberculosis* show no evidence of a memory response, suggesting a level of innate resistance that can function prior to engagement of the adaptive immune system (Cobat *et al*., 2009). We aimed to identify innate immunological mechanisms that underlie this diverse spectrum in clinical outcome (Barry *et al*., 2009). In light of the fact that natural killer (NK) cells can produce IFNγ (Schoenborn and Wilson, 2007), express cytolytic activity (Lanier *et al*., 1986), and respond to conserved determinants of microbial pathogens through Toll-like and other innate immune receptors (Chalifour *et al*., 2004), we wished to investigate whether these cells could constitute one variable in the immune response to *M. tuberculosis*.

Human NK cells display extensive phenotypic heterogeneity and plasticity within and between individuals. For instance, the level of CD56 surface expression discriminates two major subsets of NK cells whose frequencies in the blood vary significantly between individuals (Cooper *et al*., 2001). Such variation could have functional immune consequences since CD56^bright^ cells are usually associated with cytokine production, and CD56^dim^ cells with natural cytotoxicity (Poli *et al*., 2009) although this dichotomy has been recently refined (De Maria *et al*., 2011). Furthermore, each individual possesses a most likely unique repertoire of NK cells due to (i) the inherited set of genes and alleles coding for each different NK cell receptor, and (ii) the stochastic expression of these genes among NK cells from the same individual (Uhrberg, 2005). Moreover, there is growing evidence that NK cell responses are tuned by a process that involves an interaction between Killer Immunoglobulin-like Receptors (KIR) on NK cells and host-specific MHC class I molecules (Brodin *et al*., 2009). A consequence is that variations between individuals in the repertoire of KIR alleles expressed by NK cells can be associated with differences in responses to pathogen-associated signals and with resistance or susceptibility to different diseases (Parham, 2005; Kulkarni *et al*., 2008; Korbel *et al*., 2009). In the context of tuberculosis, a higher prevalence of KIR2DL3 among TB patients has been observed in two independent studies (Mendez *et al*., 2006; Mahfouz *et al*., 2011).

In a murine model of tuberculosis, NK cells were found to be recruited to the lung and to produce IFNγ and perforin, although the absence of an infection phenotype following antibody-mediated depletion led the investigators to conclude that their functional role was redundant (Junqueira-Kipnis *et al*., 2003). In experiments using γ-chain^−/−^RAG^−/−^ mice in comparison with RAG^−/−^ immunodeficient mice, significant NK cell contribution was evident to the control of *M. tuberculosis* infection in the absence of T cell function (Feng *et al*., 2006), consistent with a model in which NK cells provide an alternative source of activities overlapping with those of other immune cells. However the extent to which NK cell contribution derived from mice studies can be extended to humans is notably limited by the inherent expression of independently evolved and structurally unrelated set of MHC class I-specific NK cell receptors belonging to the C-type lectin-like family as opposed to the immunoglobulin-like family in humans (Parham, 2005).

In humans, several clinical studies have explored the potential association between peripheral blood NK cell counts and resistance or susceptibility to tuberculosis (Barcelos *et al*., 2008; Bozzano *et al*., 2009; Leung *et al*., 2009). A reduction in frequency and functionality of CD56^bright^ NK cells has been observed in patients with active tuberculosis and reciprocally, high blood levels were associated with protection in putative tuberculosis-resistant individuals. Variations in the number of NK cells in cord blood have also been suggested to influence the efficacy of BCG vaccination (Watkins *et al*., 2008; van den Biggelaar *et al*., 2009).

The present study provides the first evidence of the presence of NK cells in human granulomatous lesions showing that these cells are taking part in the immune response during pulmonary tuberculosis. The cytotoxicity against *M. tuberculosis*-infected cells has been addressed and the mechanisms characterized (Vankayalapati *et al*., 2002; Garg *et al*., 2006). However, direct IFNγ responses of peripheral blood NK cells to mycobacterial preparations focused only on attenuated strains, gamma-irradiated or heat-killed mycobacteria rather than fully virulent *M. tuberculosis* (Wang *et al*., 2004; Batoni *et al*., 2005; Schierloh *et al*., 2007). Therefore, the consequence of a direct interaction between live *M. tuberculosis* and human NK cells is still unknown. Hence, we performed a systematic analysis of the responses of NK cells from various anonymous human blood donors, comparing cytokine response intensity to extracellular virulent *M. tuberculosis* H37Rv with the response to the attenuated *Mycobacterium bovis* BCG Pasteur strain. We observed that the major determinant of the NK cell response to mycobacteria is coming from the host and is independent of mycobacterial virulence. We describe an important variation of the cytokine response intensity between NK cells from different individuals and demonstrate a correlation with KIR gene content.

## Results

### NK cells are recruited to the lungs during *M. tuberculosis* infection

Tuberculosis is generally treated by chemotherapy. However, tuberculous patients suffering from multi-drug-resistant tuberculosis may undergo surgery as an adjunctive approach to reduce disease burden, which gives access to resected lung tissue. Based on NKp46, a single universal marker for mammalian NK cells (Walzer *et al*., 2007), we used immunofluorescent microscopy to look for NK cells, screening sections from five formalin-fixed and paraffin embedded tuberculous lesions covering most of the different types of human granulomas as reviewed by Leong *et al*. (2011). We were able to detect numerous NK cells especially within inflammatory cell infiltrates in a sample representative of a necrotizing lesion (Fig. 1, panel b, c) and also within the well-vascularized fibrotic wall delimiting this granuloma (Fig. 1, panel a, d). The proximity of the NK cells to blood vessels was almost universal, suggesting recent extravasation. We observed a similar distribution within the consolidated area of a tuberculous pneumonia within the fibrotic surroundings of a large necrotizing granuloma (Fig. S1). In another sample, NK cells could be found infiltrating the epithelioid macrophage layer of a well-cuffed granuloma where liquefaction was evident, and within the chronic inflammatory area juxtaposed to it (Fig. S2). NKp46 signals were rarely observed in unaffected airway tissues and only few signals could be detected in the surroundings of a calcified granuloma or within its sclerotic rim (Fig. S3). These observations suggest that, during active tuberculosis, NK cells are recruited at the site of disease especially within highly inflamed granulomatous lesions. At this stage, NK cells can interact with infected cells and also extracellular mycobacteria released following lysis of infected cells by specific CD8 T cells or NK cells themselves.

**Figure:**
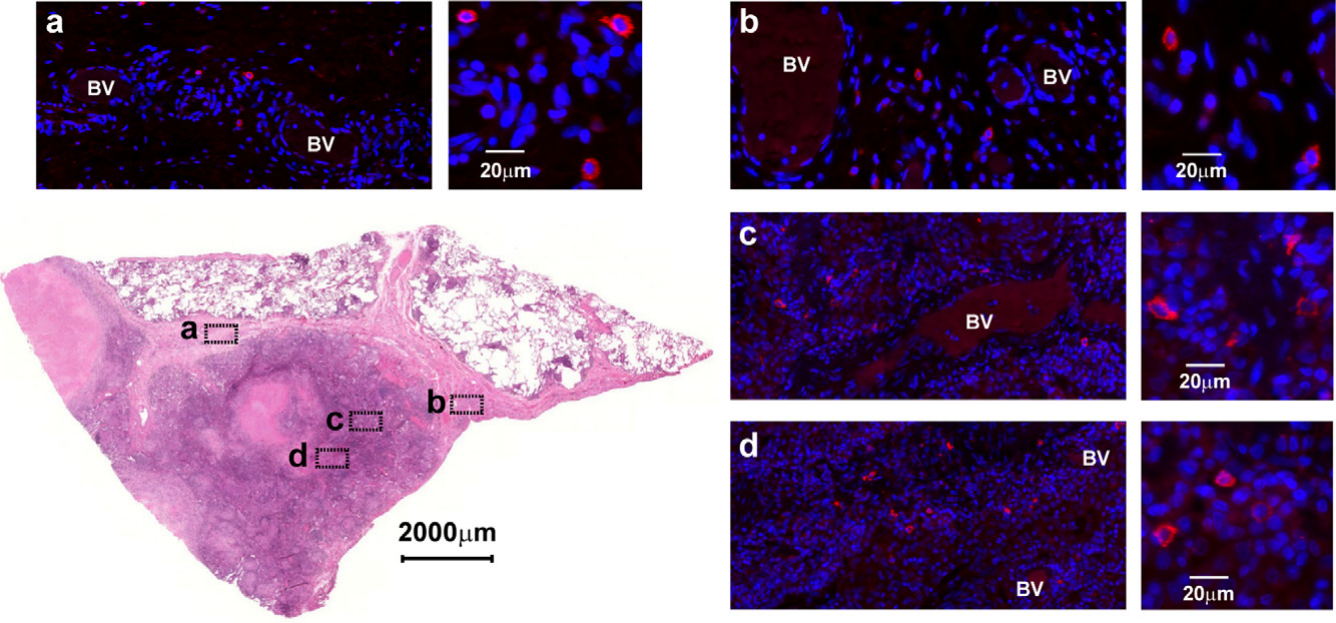
NK cells are recruited to the lungs during *M. tuberculosis* infection. Bottom left, H&E stain of a section from a necrotizing lesion resected from the lung of a tuberculous patient that was used for immunofluorescence microscopy assays. (a to d) Insets from a representative immunostained serial section showing the presence of NK cells (NKp46^+^ in red) within fibrotic and vascularized regions surrounding the necrotic centre (BV: blood vessel). Each inset shows a focus on positive cells at higher magnitude. Slides were counter-stained with DAPI (blue).

### IFNγ production by NK cells in response to extracellular mycobacteria requires cytokine co-stimulation

We aimed to study the consequences of a direct interaction between NK cells and a virulent strain of *M. tuberculosis* and to determine whether mycobacterial virulence could affect this interaction. We therefore started screening for optimal time and conditions in which NK cells would respond to mycobacterial stimulation (Fig. 2). We cultivated purified human NK cells with or without single cell suspensions of *M. tuberculosis* H37Rv or *M. bovis* BCG (MOI 1:1) in the presence or absence of two common co-stimulatory cytokines for NK cell activity (i.e. IL-2 [100 U ml^−1^) or IL-12p70 (1 ng ml^−1^)]. We collected supernatants every 24 h for 3 days and measured release of IFNγ. In this experimental setting, cytokines or mycobacteria alone were not sufficient to independently trigger IFNγ production by NK cells. However, we observed progressive accumulation of IFNγ in culture supernatants from 24 h to 48 h that began to plateau after 72 h of contact with the mycobacteria and IL-2 or IL-12p70. In both cytokine environments, the attenuated BCG vaccine strain elicited a comparable response to virulent *M. tuberculosis* H37Rv. Although the plateau value varies between donors, this kinetic pattern of IFNγ production was found consistent across three independent experiments.

**Figure:**
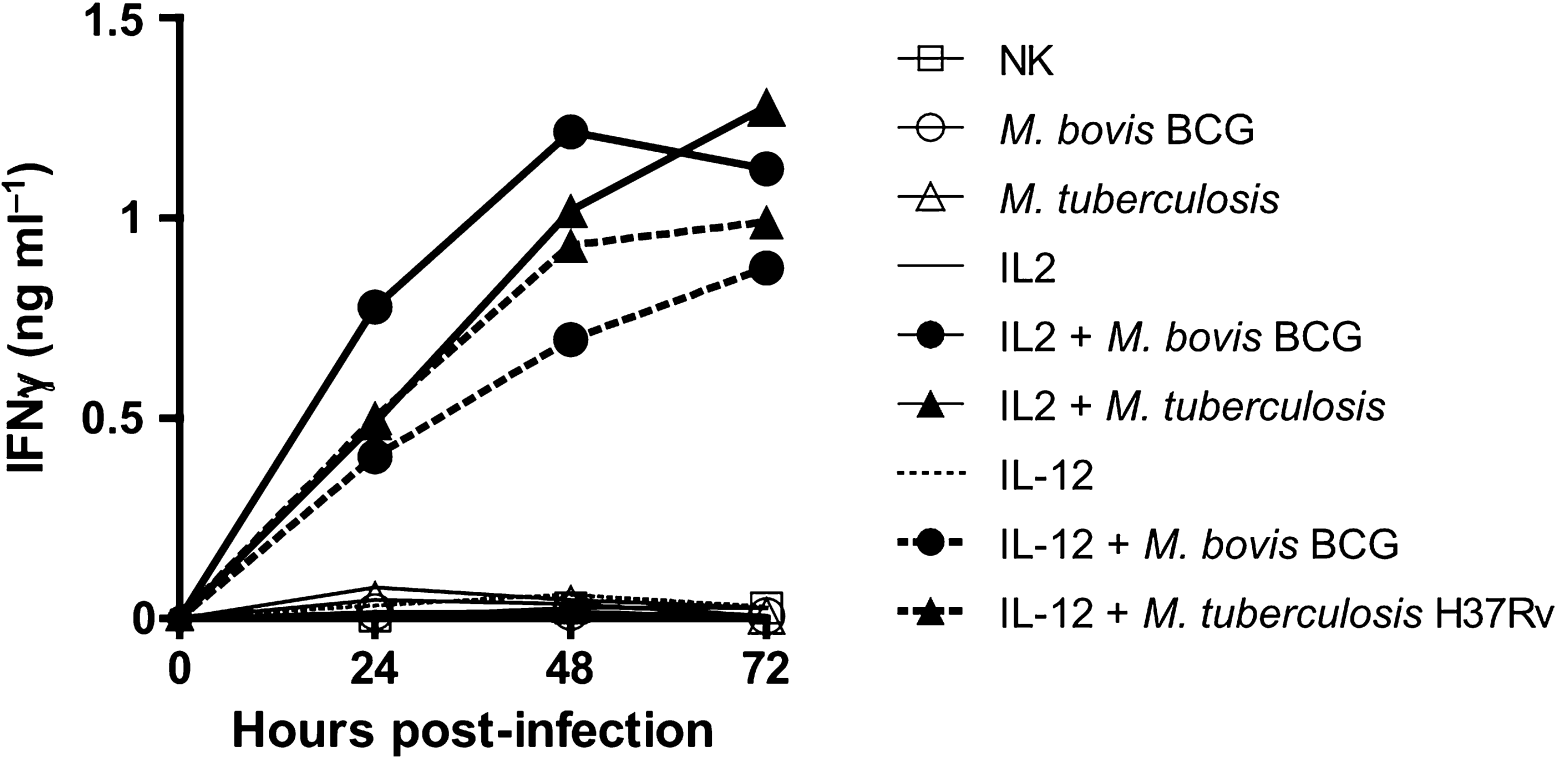
NK cell IFNγ response to mycobacteria requires cytokine stimulation. NK cells purified from human PBMCs were cultivated with or without single cell suspensions of *M. tuberculosis* H37Rv (triangles) or *M. bovis* BCG (circles) at a multiplicity of infection (MOI) of 1:1 in the presence (filled) or in the absence (opened) of IL-2 (100 U ml^−1^) (continuous lines) or IL-12p70 (1 ng ml^−1^) (dashed lines). Supernatants were collected every 24 h for 3 days and IFNγ measured by ELISA. Mycobacteria alone were not able to trigger the production of IFNγ by resting NK cells. However, NK cells released IFNγ from 24 h reaching a plateau 72 h post contact when co-activated with IL-2 or IL-12p70. *M. tuberculosis* H37Rv and *M. bovis* BCG showed comparable potency to induce the production of IFNγ by NK cells. Comparable kinetics for mycobacterial sensing by NK cells were observed across three independent separate experiments.

### IFNγ production by NK cells in response to extracellular mycobacteria is independent of mycobacterial virulence

We subsequently compared the NK cell response from three anonymous donors that were isolated, cultivated for 72 h in the presence or in the absence of mycobacteria (MOI 1:1) and/or co-stimulatory cytokines, and analysed simultaneously. Figure 3 illustrates the donor variability in the final amount of IFNγ released by NK cells following contact with mycobacteria, independently of mycobacterial strain. Indeed when looking at each donor individually, we confirmed that *M. tuberculosis* was able to trigger very similar cytokine response intensities as *M. bovis* BCG in both cytokine environments. Using intracellular antibody staining and polychromatic flow cytometry on another set of donors, we confirmed that IFNγ originated from NK cells (Fig. 4A). We also observed that the amount of IFNγ found in the supernatants after 72 h reflected the frequency of IFNγ positive NK cells 24 h post stimulation (Fig.4B).

**Figure:**
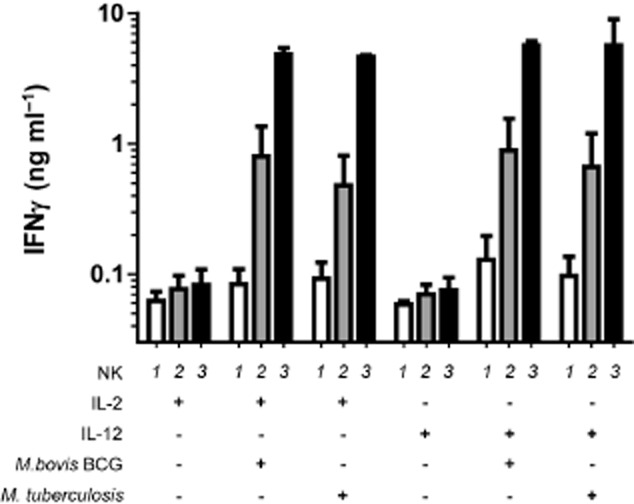
NK cell IFNγ response to mycobacteria is independent of mycobacterial virulence. NK cells were purified from three independent donors and cultured in parallel in the presence of IL-2 (100 U ml^−1^) or IL-12 (1 ng ml^−1^) and live *M. bovis* BCG or *M. tuberculosis* H37Rv (MOI 1:1). Supernatants were harvested after 72 h and assessed for IFNγ content. When looking at the cytokine response for each donor taken individually, *M. bovis* BCG shows comparable antigenicity to *M. tuberculosis*. However, the IFNγ response intensity was found very variable between the different NK cell preparations. Donors are numbered arbitrarily to be presented in ascending order of response. (One of three representative experiments)

**Figure:**
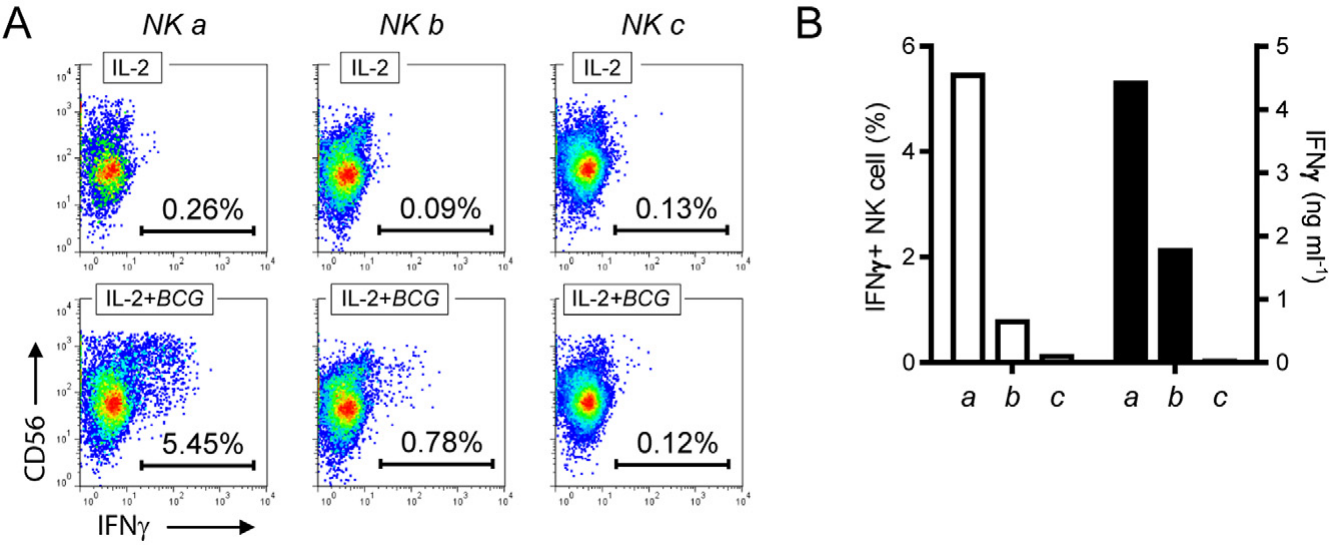
Intracellular cytokine staining of NK cells exposed to extracellular mycobacteria correlates with the amount of IFNγ detected in supernatants. NK cells were purified from three independent donors (‘a’, ‘b’ and ‘c’) and cultured in parallel in the presence of IL-2 (100 U ml^−1^) and live *M. bovis* BCG (MOI of 1:1) for 18 h before brefeldin A treatment, antibody staining and flow cytometry analysis. A. Pseudo-colour dot-plots showing variable *de novo* induction of IFNγ production by mycobacteria (lower quadrants) across the three NK cell preparations but not in the presence of IL-2 only (upper quadrants). B. Histogram comparing the frequency of IFNγ-positive NK cells after 24 h of mycobacterial exposure in the presence of IL-2 (100 U ml^−1^) (white bars) and the amount of IFNγ measured after 72 h of contact with mycobacteria and IL-2 (black bars). (One of three representative experiments)

### NK cell secretion profile after mycobacterial stimulation highlights substantial donor variability

Since our previous observations suggested quantitative differences in the predisposition of NK cells from individual anonymous donors to respond to mycobacteria, we evaluated this variability in a larger donor sample size. Using standardized culture conditions, we recorded the cytokine response of purified NK cells from 52 independent donors after 72 h of contact with mycobacteria (MOI 1:1). Since neither the mycobacterial virulence nor the nature of the co-stimulatory cytokine influenced the NK cell response in the previous experiments, we arbitrarily chose to limit this screen to *M. bovis* BCG and IL-2 (100 U ml^−1^) as co-stimulatory cytokine. As a result of the lower threshold of detection of the bead fluorescent technology used in this experiment to measure cytokine production, we could now observe a slight but significant induction of IFNγ production by IL-2 alone but not with mycobacteria (Fig.5A). IFNγ production in response to co-stimulation with IL-2 and live mycobacteria extended over three orders of magnitude when comparing the different donor responses. In addition, since a previous study suggested that *M. bovis* BCG could trigger the production of TNFα by NK cells (Marcenaro *et al*., 2008), we also measured the production of this cytokine in the same set of samples. As with IFNγ, we observed a slight but significant induction of TNFα secretion by IL-2 but also by mycobacteria alone and a synergistic effect of NK exposure to both mycobacteria and IL-2 (Fig.5B). TNFα production was also variable across the set of donors and there was a significant correlation between the ability of each independent donor to produce IFNγ and TNFα simultaneously (Spearman *r* 0.7426, *P* < 0.0001).

**Figure:**
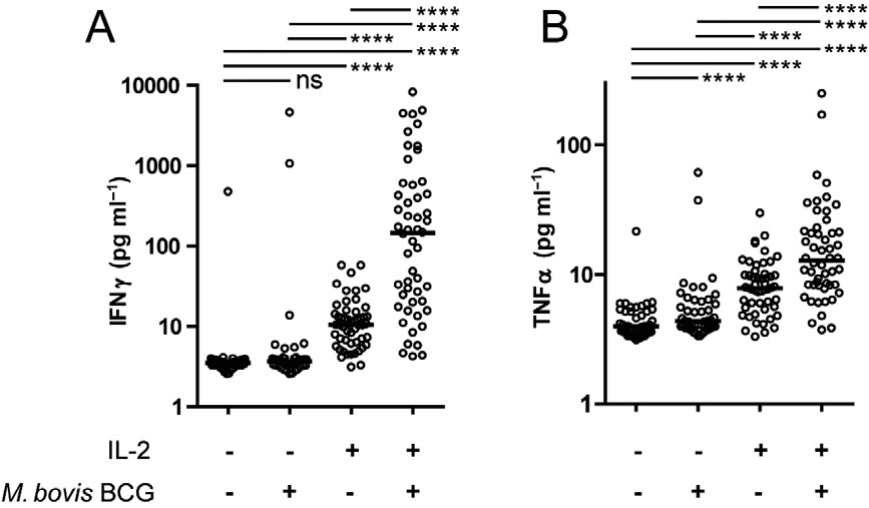
NK cell secretion profile highlights substantial donor variability. Purified NK cells from 52 independent donors were cultured in the presence or in the absence of IL-2 (100 U ml^−1^) and live *M. bovis* BCG (MOI 1:1) for 72 h. Cell-free supernatants were subjected to simultaneous multiple analytes measurement technology (A: IFNγ; B: TNFα). Direct interaction with mycobacteria in synergy with IL-2 induced the production of IFNγ and TNFα by human NK cells (Wilcoxon matched-pairs signed rank test, ****P < 0.0001). This cytokine production shows important variation across different donors. There is a statistically significant correlation between the intensity of individual IFNγ versus TNFα responses (Spearman *r* 0.7426, *P* < 0.0001).

### Mycobacteria preferentially trigger NK cell donor associated with KIR B haplotype

There is increasing evidence that different KIR/HLA genotypes influence NK cell potency and the threshold of their responsiveness (Kim *et al*., 2008; Brodin *et al*., 2009). We therefore characterized the gene content of the KIR cluster for each of the 52 donors screened in the standardized assay. This characterization was performed by PCR using two independent sets of primers per gene as previously described (Martin and Carrington, 2008) and summarized in Table 1. KIR haplotypes can be segregated in two groups (A and B) according to their gene content (Garcia *et al*., 2003). In contrast to B haplotypes, which show higher genetic diversity, the haplotype A group is characterized by the absence of *KIR2DL5*, *KIR2DS1*, *KIR2DS2*, *KIR2DS3*, *KIR2DS5* and *KIR3DS1* and would express one single activating KIR, KIR2DS4. Interestingly, we found a significantly higher proportion of donors that were homozygous for the AA haplotypes within the group of low responders, i.e. below the median response (Fig. 6). Therefore, donors harbouring one or several activating receptors other than KIR2DS4 are significantly more represented in the group of high responders. When we looked for the activating KIR that could drive this association, we found that KIR2DS3 and KIR2DS5, two similar KIR with unidentified ligands, were significantly over-represented within the group of high responders [χ^2^, d.f. (3.82, 1), *P* = 0.0253].

**Figure:**
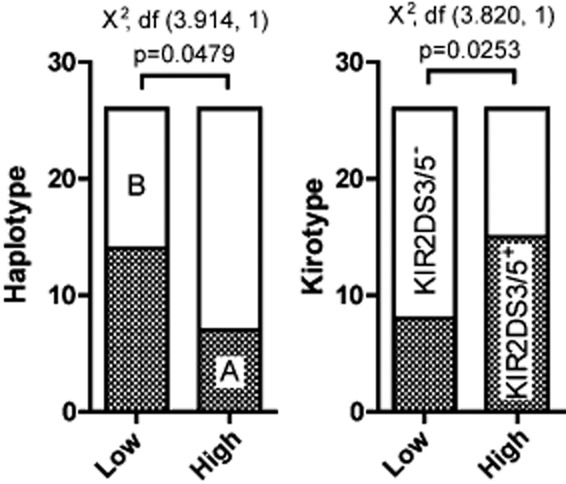
KIR B haplotype is associated with a higher IFN-gamma response to mycobacterial stimulation. A. KIR genotype frequency, extracted from Table 1 revealed a significant association between B haplotypes and high responders, defined as those above the median response of IFNγ. B. The activating receptors KIR2DS3 and KIR2DS5 were found to drive this association with a significant higher prevalence within the group of high responders (chi-squared test, *P* < 0.05).

**Table:** Analysis of the Leucocyte Receptor Complex (LRC) locus shows higher representation of KIR haplotypes B within high responders

	IFNγ	3DL3	2DL4	3DL2	2DL2	2DL3	2DL5	2DL1	3DL1	2DP1	2DS4	2DS1	2DS2	2DS3	2DS5	3DS1	Haplo
Low																	
E3	4.3	+	+	+	−	+	+	+	+	−	+	+	−	−	+	+	B
D3	4.4	+	+	+	+	−	+	+	+	+	+	−	+	+	−	−	B
H4	4.7	+	+	+	−	+	−	+	+	+	+	−	−	−	−	−	A
G4	5.8	+	+	+	−	+	−	+	+	+	+	−	−	−	−	−	A
S4	6.1	+	+	+	−	+	−	+	+	+	+	−	−	−	−	−	A
F4	8.4	+	+	+	−	+	−	+	+	+	+	−	−	−	−	−	A
M4	10.0	+	+	+	+	+	+	+	+	+	+	+	+	−	+	+	B
T4	11.1	+	+	+	−	+	−	+	+	+	+	−	−	−	−	−	A
D\4	13.5	+	+	+	+	+	−	+	+	+	+	−	+	−	−	−	B
L4	15.7	+	+	+	−	+	−	+	+	+	+	−	−	−	−	−	A
K3	15.8	+	+	+	+	+	−	+	+	+	+	−	+	−	−	−	B
E4	17.6	+	+	+	−	+	−	+	+	+	+	−	−	−	−	−	A
I4	20.0	+	+	+	−	+	−	+	+	+	+	−	−	−	−	−	A
C4	20.6	+	+	+	−	+	−	+	+	+	+	−	−	−	−	−	A
U3	24.8	+	+	+	+	+	+	+	+	+	+	+	+	+	+	+	B
R4	27.0	+	+	+	−	+	+	+	−	+	−	+	+	−	+	+	B
N4	29.8	+	+	+	+	+	+	+	+	+	+	+	+	+	−	+	B
G3	31.6	+	+	+	−	+	−	+	+	+	+	−	−	−	−	−	A
K4	33.2	+	+	+	+	+	+	+	−	+	−	+	+	−	−	−	B
J4	36.1	+	+	+	−	+	−	+	+	+	+	−	−	−	−	−	A
Q4	40.0	+	+	+	+	+	−	+	+	+	+	−	+	−	−	−	B
W3	48.9	+	+	+	−	+	−	+	+	+	+	−	−	−	−	−	A
J3	81.3	+	+	+	+	+	+	+	+	+	+	+	+	−	+	+	B
A4	95.4	+	+	+	−	+	−	+	+	+	+	−	−	−	−	−	A
O3	115.4	+	+	+	−	+	−	+	+	+	+	−	−	−	−	−	A
I3	142.5	+	+	+	−	+	+	+	+	+	+	+	−	−	+	+	B
*Frequency*		100	100	100	34.62	96.15	34.62	100	92.31	96.15	92.31	30.77	38.46	**11.54**	**23.08**	30.77	**53.85**
High																	
P3	148.9	+	+	+	+	+	−	+	+	+	+	−	+	−	−	−	B
F3	159.2	+	+	+	+	+	+	+	+	+	+	+	+	+	−	+	B
D4	160.2	+	+	+	+	+	+	+	+	+	+	−	+	+	−	−	B
Z3	172.8	+	+	+	+	+	−	+	+	+	+	−	+	−	−	−	B
V3	207.0	+	+	+	−	+	+	+	+	+	+	−	−	−	+	+	B
H3	225.9	+	+	+	−	+	−	+	+	+	+	−	−	−	−	−	A
B4	237.8	+	+	+	+	+	+	+	−	+	−	+	+	+	+	+	B
Z2	256.3	+	+	+	+	−	+	−	+	−	+	+	+	−	+	+	B
E\4	285.9	+	+	+	+	+	−	+	+	+	+	−	+	−	−	−	B
B\4	345.3	+	+	+	+	−	+	+	+	+	+	−	+	+	−	−	B
B3	399.5	+	+	+	−	+	+	+	+	+	+	−	−	−	+	−	B
A3	427.0	+	+	+	−	+	−	+	+	+	+	−	−	−	−	−	A
X3	446.6	+	+	+	−	+	+	+	+	+	+	+	−	−	+	+	B
Y3	576.2	+	+	+	−	+	+	+	+	+	+	−	−	−	+	+	B
T3	608.8	+	+	+	−	+	+	+	−	+	−	+	−	−	+	+	B
S3	640.6	+	+	+	−	+	+	+	+	+	+	+	−	−	+	+	B
U4	1207.2	+	+	+	+	+	−	+	+	+	+	−	+	−	−	−	B
Q3	1581.0	+	+	+	−	+	−	+	+	+	+	−	−	−	−	−	A
M3	1770.7	+	+	+	−	+	−	+	+	+	+	−	−	−	−	−	A
C3	1793.6	+	+	+	+	+	+	+	+	+	+	+	+	+	+	+	B
P4	2662.4	+	+	+	−	+	−	+	+	+	+	−	−	−	−	−	A
O4	3320.4	+	+	+	−	+	−	+	+	+	+	−	−	−	−	−	A
L3	4416.6	+	+	+	−	+	+	+	+	+	+	+	−	−	+	+	B
C\4	4528.6	+	+	+	−	−	+	+	+	+	+	−	−	+	−	+	B
R3	4930.4	+	+	+	−	+	−	+	+	+	+	−	−	−	−	−	A
N3	8259.7	+	+	+	+	+	+	+	+	+	+	+	+	+	+	+	B
*Frequency*		100	100	100	42.31	88.46	57.69	96.15	92.31	96.15	92.31	34.62	42.31	**26.92**	**42.31**	46.15	**26.92**
													*		*

DNA was extracted from PBMCs and KIR genotyping established as previously described (Martin and Carrington, 2008). The table summarizes the presence (+) or the absence (−) of each KIR gene within the LRC. NK cell donors were ranked (top to bottom) according to the amount of IFNγ measured after 72 h of contact with mycobacteria (MOI 1:1) in the presence of IL-2 (100 U ml^−1^). Using the median, two groups referred as low versus high responders were created for subsequent statistical analysis. Frequency for each gene or haplotype within each group has been calculated and significant changes are highlighted in bold (^*^P < 0.05).

## Discussion

Consideration of the potential contribution of NK cells is generally restricted to the early phase of mycobacterial infection, prior to engagement of adaptive immunity. Our demonstration of the presence of NK cells within mature granulomatous lesions is consistent with an involvement that extends into later stages of mycobacterial pathogenesis. Given their rapid turnover (< 15 days) (Zhang *et al*., 2007), the presence of NK cells in lesions suggests constant recruitment, which is reflected by the frequency of positive signals in the vicinity of blood vessels proximal to the lesions. Our observations suggest that NK cells are frequently recruited into inflamed tissues, patrolling the lesions where they could intercept infected cells migrating out of the granuloma as well as interact with extracellular mycobacteria released after lysis by cytotoxic cells. Therefore, variation in the host genetics that could affect NK cell responses would give rise to different levels of innate resistance to *M. tuberculosis* infection.

We found that human peripheral blood NK cells have the potential to produce IFNγ when they encounter *M. bovis* BCG as well as *M. tuberculosis* in an appropriate cytokine milieu. This indicates that priming of NK cells by cytokines is sufficient to render NK cells responsive to mycobacteria. Release of IL-12 by macrophages and dendritic cells could therefore promote NK cell-mediated production of IFNγ during the early phase of infection, while IL-2 from T cells provides a potential stimulus for activation of NK cells at later stages. The fact that mycobacteria synergize with co-stimulatory cytokines to induce IFNγ production by NK cells is fully in line with previous reports showing the requirement of co-stimulatory cytokines following Toll-Like Receptor agonist stimulation (Chalifour *et al*., 2004; Souza-Fonseca-Guimaraes *et al*., 2012). We did not perform an exhaustive screen of potential co-stimulatory cytokines but it is very likely that IL-15 among other cytokines may also synergize with the stimulation triggered by mycobacteria as observed for Pathogen-Associated Molecular Pattern recognition. Moreover, it has been shown that a cellular contact with APCs can modulate the cytokine response of NK cells that are recruited at the site of tuberculous pleurisy (Schierloh *et al*., 2007). Initial studies have described partial involvement of TLR2 and the natural cytotoxicity receptor NKp44 in recognition of *M. bovis* BCG (Esin *et al*., 2008; Marcenaro *et al*., 2008). The fact that we observed similar induction of IFNγ production suggests that recognition of virulent *M. tuberculosis* and attenuated BCG vaccine by human NK cells are most likely controlled by the same set of pathogen receptors.

In contrast, the level of IFNγ production by NK cells in response to mycobacterial stimulation varies over a 1000-fold between donors. Preferential expansion of particular NK cell subsets during viral infection has been shown to establish a long-lived protective memory response in mice (Sun *et al*., 2009), and it is attractive to speculate that mycobacterial infection or BCG vaccination could similarly establish an NK memory response. Having used anonymous blood donors, and therefore in the absence of medical records, we could not directly correlate NK cell responsiveness with BCG vaccination or previous *M. tuberculosis* infection for instance. However, using *M. tuberculosis* Protein Purified Derivative (PPD) to stimulate PBMCs from our panel of anonymous donors and IFNγ release assay as a read-out, we did not find any significant correlation between the NK cell responsiveness and memory immune response indicative of previous exposure to mycobacteria (Fig. S4). However, we cannot rule out the possibility of bystander amplification or modulation of NK cell activity by other infections (Marras *et al*., 2011).

A major component of NK cell inter-individual variability is associated with the expression of specific HLA/KIR haplotypes revealed as a crucial determinant of NK cell responsiveness to tumour cell lines (Kim *et al*., 2008) and pathogen-associated signals (Korbel *et al*., 2009). We have shown here that KIR B haplotypes, i.e. those harbouring multiple activating KIRs, and especially KIR2DS3 or KIR2DS5, correlates with a higher responsiveness to extracellular mycobacteria. These two receptors are similar to each other and together form the activating lineage III KIR (Moesta *et al*., 2010). Unlike other activating KIR, KIR2DS3 and KIR2DS5 were not derived from a paired inhibitory receptor by recombination and their ligands still remain unknown (Cheent and Khakoo, 2009). Our observations suggest a link between KIR genotype and the ability of the respective NK cell repertoire to react to mycobacteria. However, despite being statistically significant, the contingency test performed to study this association has limited statistical power. For instance, the use of the median as a cut-off to distinguish low from high responders has been performed arbitrarily in order to use all the collected data but other grouping would have led to different interpretations. Sample size calculation indicates that further study would require a much bigger sample size to fully attest the link between KIR haplotype and the cytokine response to *M. tuberculosis*. For instance, 2236 samples for each arm should be screened in order to detect a difference of 160 units of IFNγ on average between each haplotype group (5% significance level, 80% power). It also important to highlight the fact that KIR haplotype does not fully segregate low from high responders and some NK cell preparation from KIR A individuals showed high IFNγ response indicating that KIR haplotype is certainly not the only determinant of the cytokine response to *M. tuberculosis*. Still, this donor variability highlights a substantial potential impact on the pathogenesis of *M. tuberculosis* that has to be considered. One could argue that higher IFNγ production should confer a better protection against an intracellular pathogen through macrophage activation, although higher inflammation could also contribute to exacerbated tissue destruction that is required for transmission in tuberculosis. Three published studies have addressed the influence of KIR genotype in tuberculosis in Mexican, Lebanese and Iranian populations respectively (Mendez *et al*., 2006; Mahfouz *et al*., 2011; Tajik *et al*., 2012). In the Lebanese study, KIR A haplotype was found more prevalent in the group of tuberculosis patients when compared with healthy controls (2.6:1 versus 1.5:1). However, no association could be found in the Iranian study. Therefore, the relations between NK cell repertoire, mycobacterial responsiveness and the protection or the sensitivity to tuberculosis need to be tested in various population settings.

To conclude, our results contribute to the growing evidence that NK cell activities are regulated by a complex interplay between multiple stimulatory and inhibitory signals which generates extensive functional diversity in NK cell populations between individuals (Boyton and Altmann, 2007; Kim *et al*., 2008). Thus, with their ability to deliver a range of functions that complement various aspects of innate and adaptive immunity, NK cells may make an important contribution to diversity of the human immune response to tuberculosis. Efforts to identify correlates of immune susceptibility to tuberculosis and indicators of successful vaccination are therefore likely to be enhanced by evaluation of NK cell responses alongside measurement of T cell markers in immunological analyses.

## Experimental procedures

### Ethics statement

The tissues used in this study were collected between 2003 and 2006 as previously described (Leong *et al*., 2011) with written consent of the subjects, approval of the National Masan Tuberculosis Hospital IRB and an exemption by the US NIH, Office of Human Subjects Research.

### Blood samples, cells and cell cultures

Fresh blood packs (Buffy coats and Cones) from healthy adult donors were purchased anonymously from National Blood Services, London, UK. Peripheral blood mononuclear cells (PBMCs) were prepared on a Ficoll-Paque density gradient (Amersham Biosciences AB, Uppsala, Sweden) by centrifugation (800 g, 30 min at room temperature), washed twice and frozen in RPMI 1640-FCS (5%)-DMSO (8.7%)-methyl-cellulose (0.1%). NK cells were selected from PBMCs by magnetic cell sorting using indirect NK isolation kit (Miltenyi Biotec, Auburn, CA, USA) according to manufacturer's recommendations. Average NK cell purity checked by flow cytometry (CD3^−^/CD16^+/−^/CD56^+/−^) was 97.51% ± 2.47 (standard deviation) across all donors. NK cells were cultured in complete RPMI 1640 medium, including 1 mM sodium pyruvate, and 1% heat-inactivated fetal calf serum. Recombinant human IL-2 and IL-12 were obtained from PeproTech EC.

### Culture and preparation of mycobacterial strains

*Mycobacterium tuberculosis* H37Rv and *M. bovis* BCG Pasteur were grown at 37°C in Middlebrook 7H9 broth supplemented with ADC (Becton Dickinson, Sparks, USA). The strains were grown to mid-exponential growth phase and pelleted at room temperature. Single cell bacterial suspensions were then prepared as previously described (N'Diaye *et al*., 1998). Briefly, the medium was discarded, bacteria were dispersed by shaking for 1 min with glass beads (3 mm diameter), and resuspended in PBS, pH 7.4. The remaining clumps were removed by centrifuging the supernatant for 10 min at 200 *g*. Bacteria were then plated on Middlebrook 7H11 agar plates supplemented with OADC (Becton Dickinson, Sparks, USA) to establish precise bacterial counts before and after freezing aliquots with glycerol (5% final v/v) and storage at −80°C.

### Cytokine production analysis

Cell culture supernatants were filtered using 0.2 μm 96-well filter plates (Corning) before detection of cytokines and chemokines using either ELISA kits (Peprotech) or combined cytokine singleplex assays (Invitrogen) on a Luminex^100^, χMAP^TM^ Technology. For intracellular cytokine detection, NK cells were stimulated for 24 h. Brefeldin A (BioLegend) was added during the last 6 h of culture before harvesting cells for antibody staining with anti-CD16-FITC (Miltenyi Biotec) and anti-CD56-PC7 (BD Bioscience), then fixed and permeabilized using BD Cytofix/Cytoperm™ buffer and stained with anti-IFNγ-PE (BD Biosciences). Cells were run on a BD Biosciences FACSCalibur flow cytometer and data analysed using FlowJo 7.6.4.

### Immunohistochemistry

Formalin fixed, paraffin embedded lung specimens obtained from lung resection surgery of TB patients with chronic MDR-TB were provided by the Tuberculosis Research Section of the Laboratory of Clinical Disease, NIAID, NIH, Bethesda, MD, directed by Dr Clifton E. Barry, III. Tissue sections (5 μm) were deparaffinized and rehydrated before high temperature antigen retrieval (citric acid 10 mM, pH 6) followed by a 20 min blocking step in PBS/0.15% BSA/0.1% Tween20 and further blocking with Fc Receptor Block solution (Innovex Biosciences) according to manufacturer's recommendations. Sections were then incubated overnight at 37°C with anti-human NKp46/NCR1 MAb (MAB 1850 & AF1850, R&D systems) (5 μg ml^−1^) followed by 2 h with Alexa Fluor® 594 chicken anti-mouse and donkey anti-goat IgG (Invitrogen) (20 μg ml^−1^) at room before mounting with DAPI (Vectashield). Sections were washed with PBS between each step. Bright-field and fluorescence image acquisition was performed using a Mirax MIDI Scan with an HXP120 lamp (Zeiss) and a HV-F22 camera (Hitachi). Annotated scaled images were converted to TIFF files using Mirax viewer software.

### Kirotyping

DNA was extracted from 5·10^6^ PBMCs using QIAmp DNA mini kit (Qiagen). Oligonucleotide sequences and PCR amplifications were performed as previously described (Martin and Carrington, 2008).

### Statistical analysis

Data analysis, correlation, paired *t*-tests, and contingency tests were performed using GraphPad Prism software. Without assuming a pre-defined distribution of the response tested, non-parametric statistical analysis has been used all across the study. Unless the direction of the association was expected prior to performing the assays, KIR2DS3/5 association study for instance, two-tailed statistical test were always performed. Statistical analysis of the KIR association study has been subjected to external review to the UCL statistical support services who performed sample size calculation.

## Supporting Information

Fig. S1 NK cells presence in a tuberculous pneumonia sample. At the centre, H&E stain of a section from a tuberculous pneumonia sample resected from the lung of a tuberculous patient that was used for immunofluorescence microscopy assays. Insets from a representative immunostained serial section showing the presence of NK cells (NKp46^+^ in red) in various part of the lesion, within the consolidated area (top left), within lymphoid aggregates filling alveolar spaces (lower right) and at the border of a well-delimitated necrotic lesion (upper right).

Fig. S2. NK cells presence in a consolidated and necrotizing tuberculous lesion. At the centre, H&E stain of a section from a tuberculous necrotizing granuloma that was used for the following immunofluorescence microscopy assays. Insets from a representative immunostained serial section showing the presence of NK cells (NKp46^+^ in red) starting notably to infiltrate the epithelioid macrophage layer delimiting a large necrotic lesion.

Fig. S3. NK cells localization in a calcified tuberculous lesion. Top left, H&E stain of a section of a calcified granuloma that was used for the following immunofluorescence microscopy assays. Insets from a representative immunostained serial section reveal the presence of NK cells (NKp46^+^ in red) recently extravasated from a blood vessel (BV) (inset b), or within surrounding alveolar spaces (inset a, d) of a calcified granuloma. Few signals could be detected at the periphery and infiltrating the sclerotic rim (inset c).

Fig. S4. PBMCs immune response intensity following exposure to *M. tuberculosis* antigens does not correlate with the ability of respective NK cells to respond to mycobacteria. Histogram comparing IFNγ production from (i) PBMC stimulated with Purified Protein Derivative (PPD) from *M. tuberculosis* (2 μg ml^−1^) for 24 h and (ii) NK cell preparation from the matching donor following exposure to *M. bovis* BCG for 72 h (MOI 1:1) in the presence of IL-2 (100 U ml^−1^). We observed substantial differences in the intensity of the immune response to PPD among the 52 donors (28 donors below 20 pg ml^−1^, 16 donors comprised between 20 and 100 pg ml^−1^ and 8 donors over 100 pg ml^−1^). However, there was no evident correlation between the memory response to PPD among PBMCs and the variable responsiveness of NK cell exposed to mycobacteria in the presence of co-stimulatory cytokine.
