# Supplementary figures and images for: Natural killer cells are recruited during pulmonary tuberculosis and their ex vivo responses to mycobacteria vary between healthy human donors in association with KIR haplotype
Source: Cell Microbiol. 2012 Jul 30;14(11):1734–44. doi: 10.1111/j.1462-5822.2012.01834.x (PMC3503254; doi:10.1111/j.1462-5822.2012.01834.x)

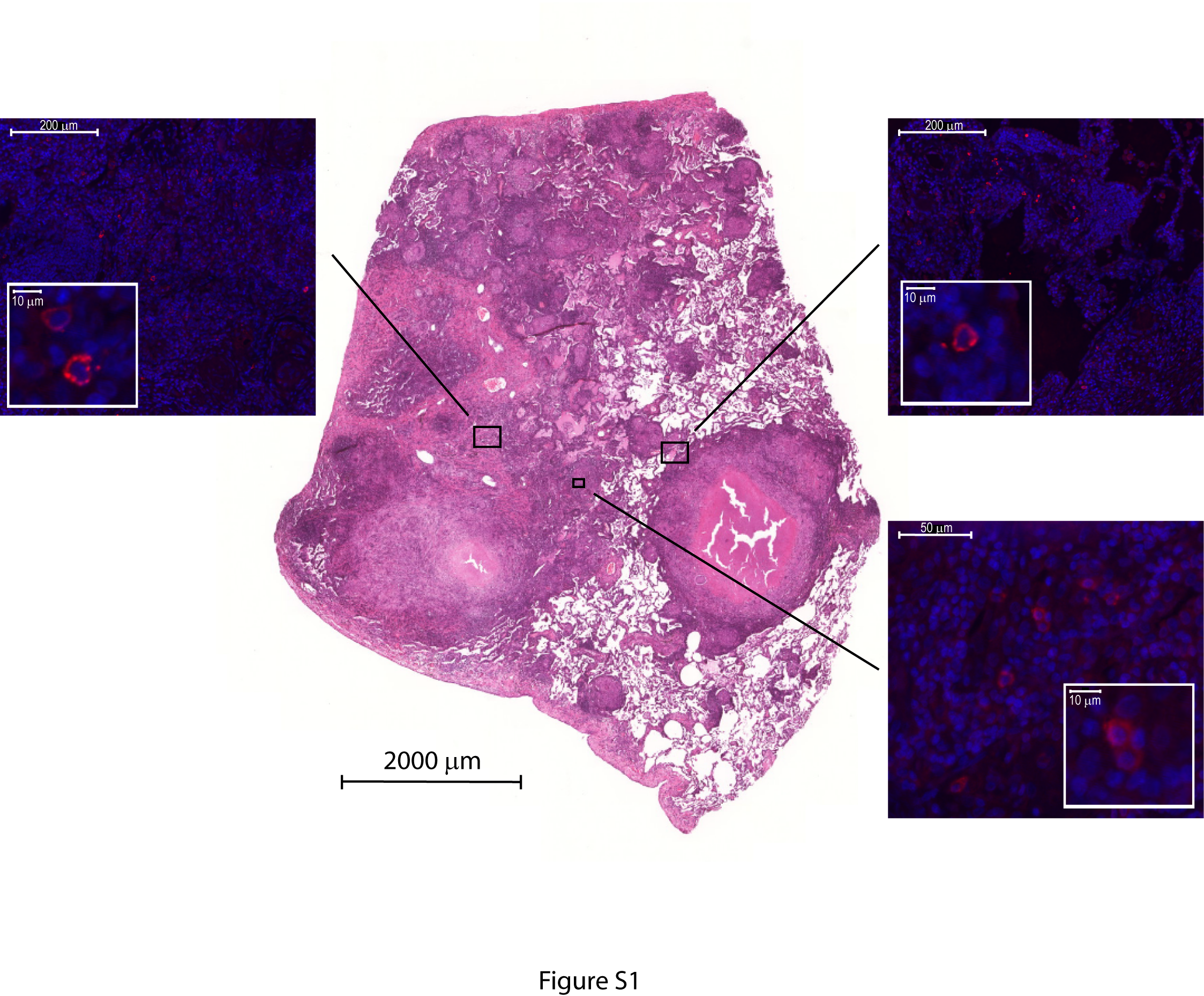

Supplement: Fig. S1 — NK cells presence in a tuberculous pneumonia sample. At the centre, H&E stain of a section from a tuberculous pneumonia sample resected from the lung of a tuberculous patient that was used for immunofluorescence microscopy assays. Insets from a representative immunostained serial section showing the presence of NK cells (NKp46+ in red) in various part of the lesion, within the consolidated area (top left), within lymphoid aggregates filling alveolar spaces (lower right) and at the border of a well-delimitated necrotic lesion (upper right). [file cmi0014-1734-SD1.tif]

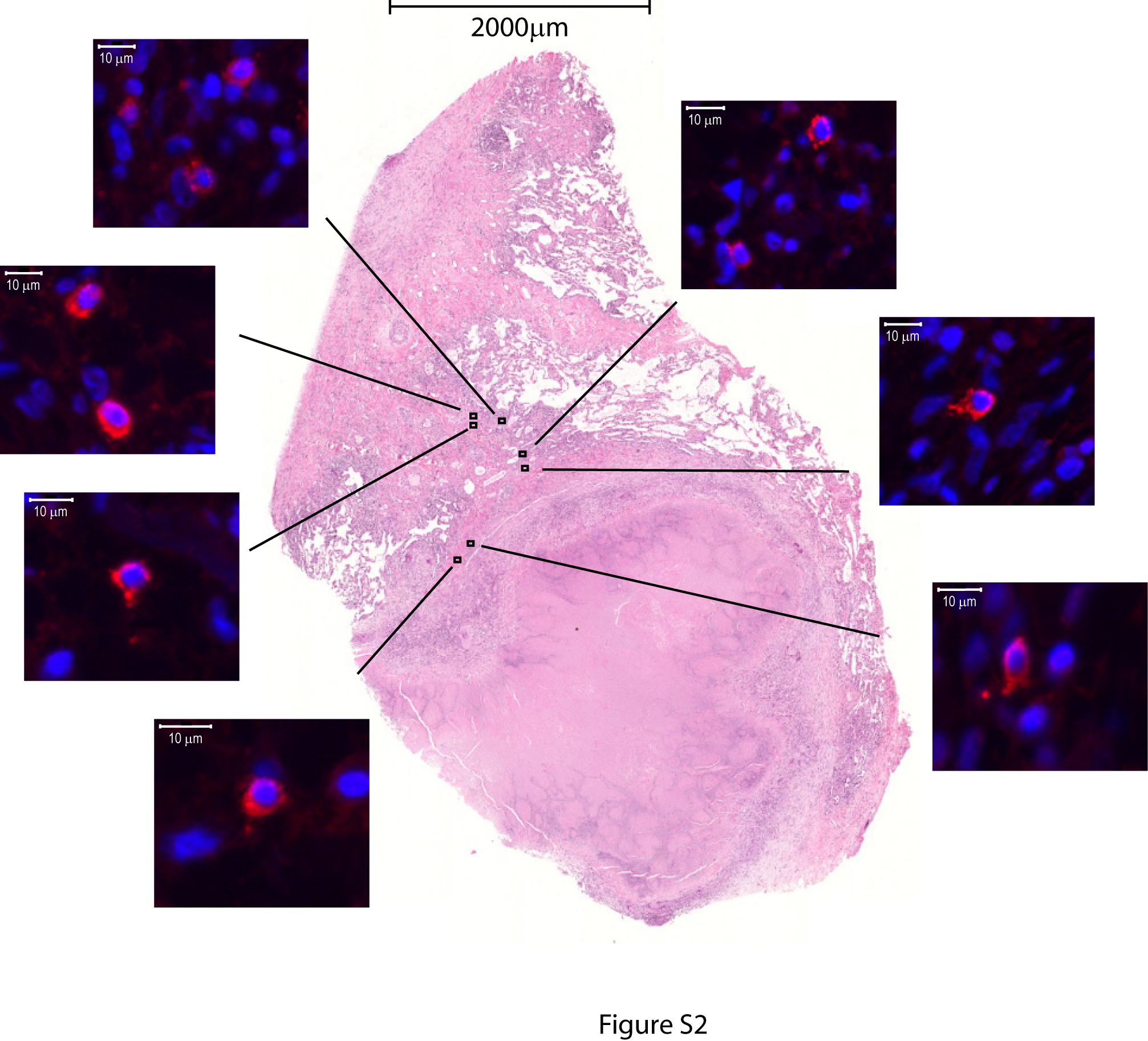

Supplement: Fig. S2. — NK cells presence in a consolidated and necrotizing tuberculous lesion. At the centre, H&E stain of a section from a tuberculous necrotizing granuloma that was used for the following immunofluorescence microscopy assays. Insets from a representative immunostained serial section showing the presence of NK cells (NKp46+ in red) starting notably to infiltrate the epithelioid macrophage layer delimiting a large necrotic lesion. [file cmi0014-1734-SD2.tif]

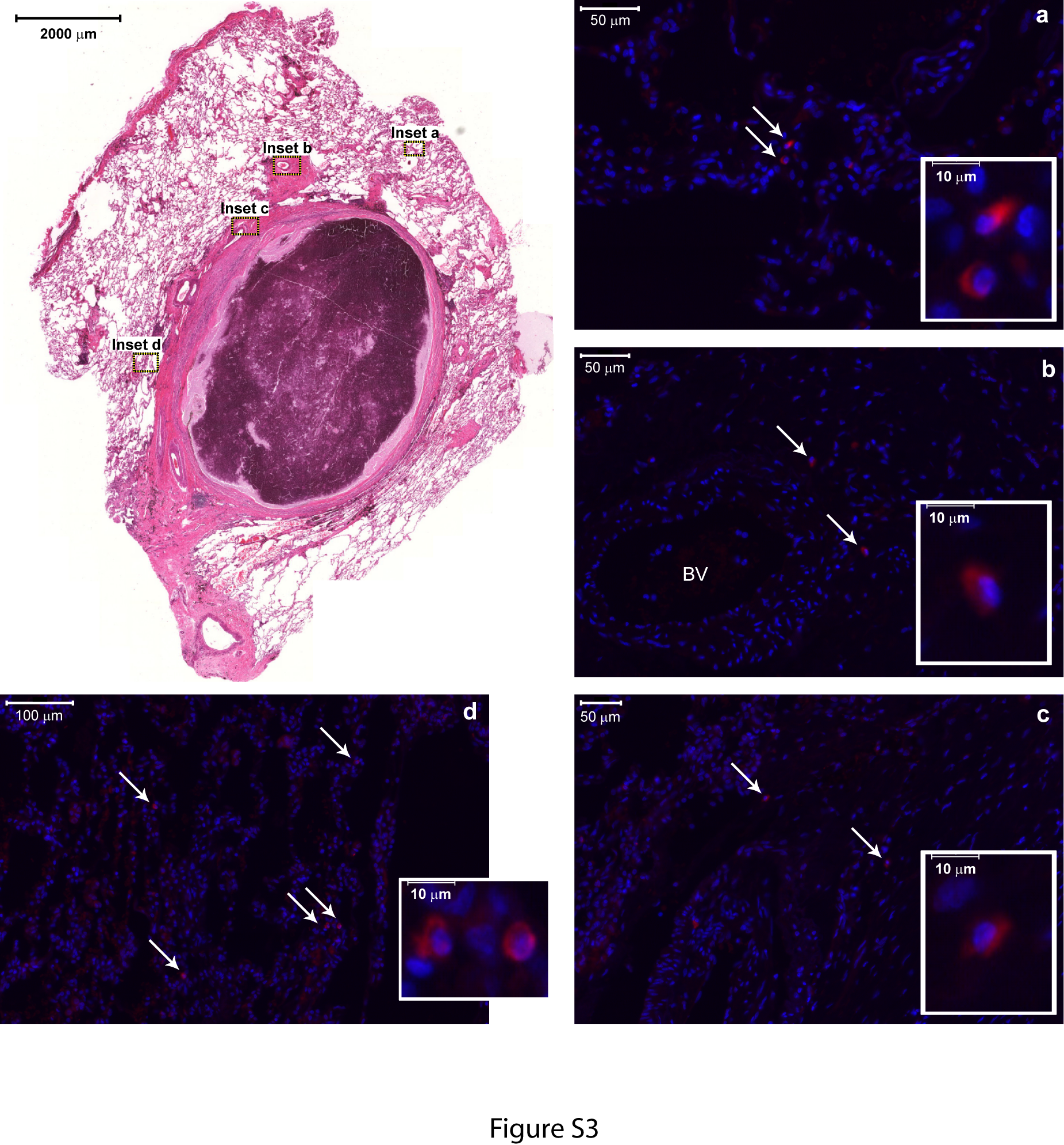

Supplement: Fig. S3. — NK cells localization in a calcified tuberculous lesion. Top left, H&E stain of a section of a calcified granuloma that was used for the following immunofluorescence microscopy assays. Insets from a representative immunostained serial section reveal the presence of NK cells (NKp46+ in red) recently extravasated from a blood vessel (BV) (inset b), or within surrounding alveolar spaces (inset a, d) of a calcified granuloma. Few signals could be detected at the periphery and infiltrating the sclerotic rim (inset c). [file cmi0014-1734-SD3.tif]

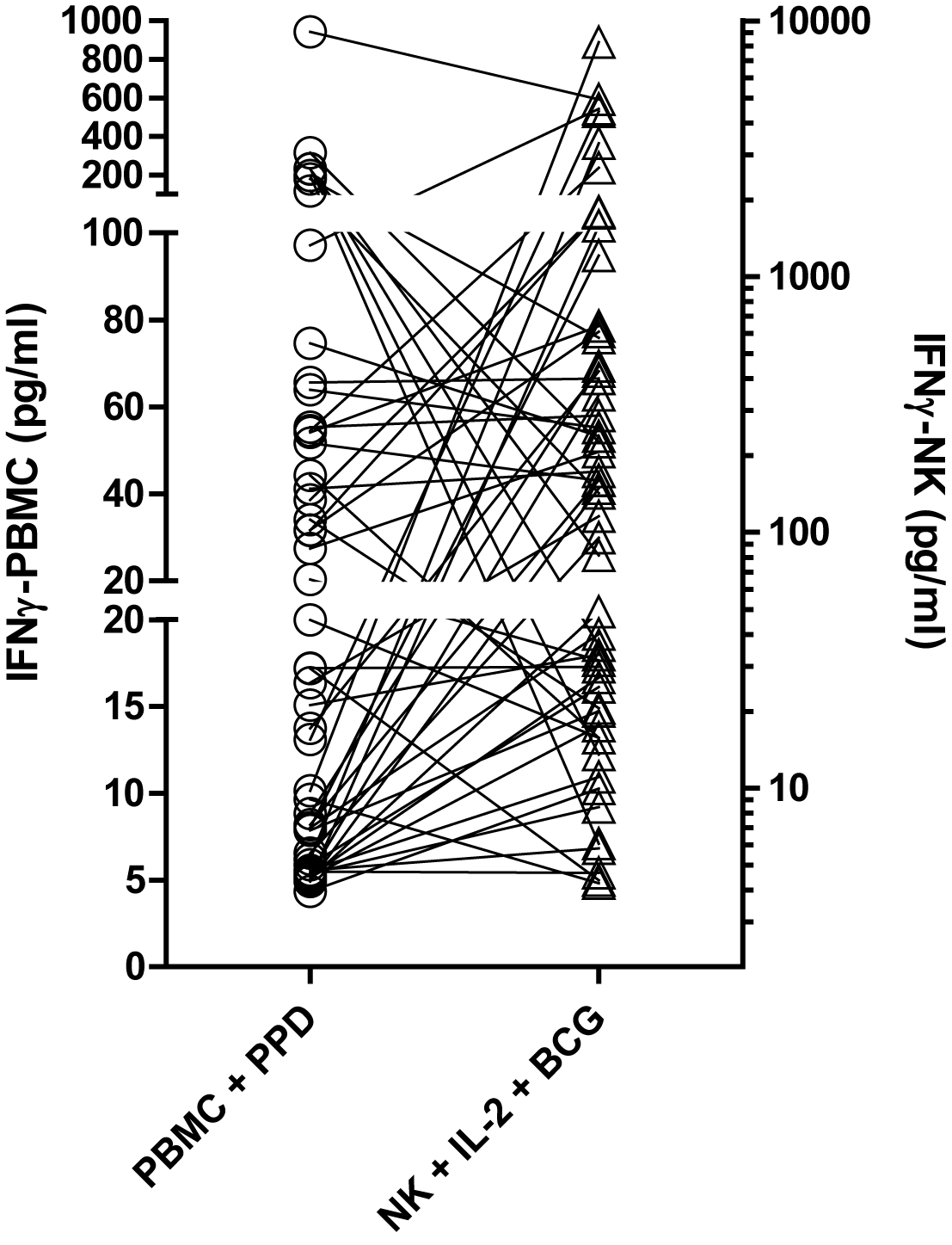

Supplement: Fig. S4. — PBMCs immune response intensity following exposure to M. tuberculosis antigens does not correlate with the ability of respective NK cells to respond to mycobacteria. Histogram comparing IFNγ production from (i) PBMC stimulated with Purified Protein Derivative (PPD) from M. tuberculosis (2 μg ml−1) for 24 h and (ii) NK cell preparation from the matching donor following exposure to M. bovis BCG for 72 h (MOI 1:1) in the presence of IL-2 (100 U ml−1). We observed substantial differences in the intensity of the immune response to PPD among the 52 donors (28 donors below 20 pg ml−1, 16 donors comprised between 20 and 100 pg ml−1 and 8 donors over 100 pg ml−1). However, there was no evident correlation between the memory response to PPD among PBMCs and the variable responsiveness of NK cell exposed to mycobacteria in the presence of co-stimulatory cytokine. [file cmi0014-1734-SD4.tif]
